# Genomic Analysis of *Melioribacter roseus,* Facultatively Anaerobic Organotrophic Bacterium Representing a Novel Deep Lineage within Bacteriodetes/Chlorobi Group

**DOI:** 10.1371/journal.pone.0053047

**Published:** 2013-01-02

**Authors:** Vitaly V. Kadnikov, Andrey V. Mardanov, Olga A. Podosokorskaya, Sergey N. Gavrilov, Ilya V. Kublanov, Alexey V. Beletsky, Elizaveta A. Bonch-Osmolovskaya, Nikolai V. Ravin

**Affiliations:** 1 Centre ‘Bioengineering’, Russian Academy of Sciences, Moscow, Russia; 2 Winogradsky Institute of Microbiology, Russian Academy of Sciences, Moscow, Russia; Université Claude Bernard - Lyon 1, France

## Abstract

*Melioribacter roseus* is a moderately thermophilic facultatively anaerobic organotrophic bacterium representing a novel deep branch within Bacteriodetes/Chlorobi group. To better understand the metabolic capabilities and possible ecological functions of *M. roseus* and get insights into the evolutionary history of this bacterial lineage, we sequenced the genome of the type strain P3M-2^T^. A total of 2838 open reading frames was predicted from its 3.30 Mb genome. The whole proteome analysis supported phylum-level classification of *M. roseus* since most of the predicted proteins had closest matches in *Bacteriodetes*, *Proteobacteria*, *Chlorobi, Firmicutes* and deeply-branching bacterium *Caldithrix abyssi*, rather than in one particular phylum. Consistent with the ability of the bacterium to grow on complex carbohydrates, the genome analysis revealed more than one hundred glycoside hydrolases, glycoside transferases, polysaccharide lyases and carbohydrate esterases. The reconstructed central metabolism revealed pathways enabling the fermentation of complex organic substrates, as well as their complete oxidation through aerobic and anaerobic respiration. Genes encoding the photosynthetic and nitrogen-fixation machinery of green sulfur bacteria, as well as key enzymes of autotrophic carbon fixation pathways, were not identified. The *M. roseus* genome supports its affiliation to a novel phylum *Ignavibateriae*, representing the first step on the evolutionary pathway from heterotrophic ancestors of Bacteriodetes/Chlorobi group towards anaerobic photoautotrophic *Chlorobi*.

## Introduction

Members of the phylum *Chlorobi*, also known as green sulfur bacteria, are strictly anaerobic obligately photoautotrophic Bacteria, which obtain electrons for anaerobic photosynthesis from sulfide and other reduced sulfur compounds [1]; [2]. The specific light-harvesting complexes, or chlorosomes, of these bacteria are large structures, consisting of bacteriochlorophylls and carotenoids [3]. Other characteristic properties of *Chlorobi* are their ability to fix nitrogen via molybdenum-containing nitrogenase [4], and to fix CO_2_ via the reverse tricarboxylic acid (TCA) cycle [5]; [6]. Bacteria from the phylum *Chlorobi* occupy a narrow environmental niche in anoxic aquatic or terrestrial environments where both sulfide and light occur, such as chemocline regions in stratified lakes [1]. Phylogenetically cultured representatives of *Chlorobi* phylum form one distinct monophyletic group that shares a common root with the *Bacteroidetes* [7]. Currently, *Bacteroidetes* and *Chlorobi* are recognised as two different phyla, but they are closely related in phylogenetic trees based on 16S rRNA as well as other gene sequences [8]; [9]; [10]. Conserved indels in several conserved proteins (FtsK, UvrB and ATP synthase α subunit) also strongly indicate that these two phyla shared a common ancestor distinguished from other bacteria [10]; [11].

Unlike *Chlorobi*, bacteria from the *Bacteroidetes* phylum (previously known as the Cytophaga-Flavobacteria-Bacteroides) are metabolically diverse chemoorganotrophes that are able to grow on a variety of complex biopolymers, such as cellulose, chitin and agar [12]; [13]. *Bacteroidetes* inhabit diverse habitats including the oral cavity and gastrointestinal tract of humans, where they represent one of the major components of its microbiome. Some species are parasites or sybmionts of humans, animals, algae and protozoa. The free-living *Bacteroidetes* inhabit soils, fresh and marine water, sediments, and a number of other mostly mesophilic environments [13].

Recently, two non-photosynthetic bacteria related to the *Chlorobi* phylum were described, - the *Ignavibacterium album* strain Mat9-16^T^ [14] and the *Melioribacter roseus* strain P3M-2^T^ [15]. The strains were isolated from microbial mats developing in streams of hydrothermal water at Yumata, Japan, and the Tomsk region of Russia, respectively. Described as moderately thermophilic, facultatively anaerobic heterotrophic bacteria, these two species and related environmental clones form a separate deep branch within the Bacteriodetes/Chlorobi group. Thereby, the novel class *Ignavibacteria* within the *Chlorobi* phylum was proposed [14]. Subsequent phylogenetic analysis, as well as chemotaxonomic and physiological studies of *M. roseus* and *I. album* suggested that these two organisms may represent a novel phylum *Ignavibacteriae* [15]. The complete genome sequence of *I. album* was recently published [16], and the analysis indicated that *I. album* is capable of organoheterotrophy under both oxic and anoxic conditions.

Here, the complete genome sequence of *M. roseus* strain P3M-2^T^ is reported. A whole genome analysis and metabolic reconstruction not only provides insight into the lifestyle of *M. roseus,* revealing its potential for polysaccharide degradation and catabolic versatility, but also supports the phylum level classification of *Ignavibacteriae*.

## Materials and Methods

### Genome Sequencing

A *M. roseus* genomic DNA sample was isolated according to Perevalova et al. [17]. The genome was sequenced on a Roche GS FLX genome sequencer using the Titanium protocol for a shotgun genome library using titanium chemistry. The GS FLX run (1/4 plate) resulted in the generation about 114 Mb of sequences with an average read length of 403 bp. The GS FLX reads were assembled into 42 contigs by a GS De Novo Assembler (Roche). The contigs were oriented into scaffolds, and the complete genome sequence was obtained upon the generation and sequencing of appropriate PCR fragments. The assembly of the genome at sites with repeated elements was verified by PCR amplification and sequencing of these regions.

### Genome Annotation and Analysis

The rRNA genes were identified with RNAmmer [18]. Transfer RNA (tRNA) genes were located with tRNAscan-SE [19]. Protein-coding genes were identified by the GLIMMER gene finder [20]. Whole-genome annotation and analysis were performed with the AutoFACT annotation tool [21]. Clusters of regularly interspaced repeats (CRISPR) were identified by CRISPR Finder [22]. Signal peptides were predicted with SignalP v. 3.0 [23]. The annotated genome sequence of *M. roseus* has been deposited in the GenBank database under accession number CP003557.

### Proteome Composition and Phylogenetic Analysis

A comparison analysis was performed for the predicted proteins encoded by the *M. roseus* genome using BLASTP to query against the NCBI non-redundant database. Those proteins that had a significant BLAST hit (e-value: 1e–05) were recorded and divided into two different categories: proteins that had a putative assigned function, and those that matched to hypothetical proteins. Proteins that did not have a significant BLAST hit were designated as species-specific proteins.

The following 39 ribosomal proteins were used in phylogenetic analysis: L20, L17, S4, S11, S13, L15, S5, L18, L6, S8, S14, L5, L24, L14, S17, L16, S3, L22, S19, L4, L3, S10, S7, S12, L7/L12, L10, L1, L11, L19, S16, L13, S9, S15, L31, L9, S18, S6, L27, and L21. Concatenated sequences of the ribosomal proteins were aligned using MUSCLE included in MEGA 5.05 [24]. Ambiguously aligned sites have been removed using Gblocks [25] before the phylogenetic reconstruction. The maximum likelihood phylogenetic trees were computed by RAxML v7.2.5 [26], using gamma model of rate heterogeneity (4 discrete rate categories, an estimated alpha-parameter) and JTT substitution matrix. The support values for the internal nodes were estimated from 100 bootstrap replicates.

### Detection of Hydrolytic Activities in vitro

The presence of endoglucanases and their approximate molecular weights were determined by zymography. Culture broths of *M. roseus*, grown on carboxymethyl cellulose (CMC) under aerobic and anaerobic conditions, were centrifuged at 12,100×*g* for 15 minutes. The pellets were rinsed twice with 50 mM Tris-HCl pH 7.7 following by centrifugation at 12,100×*g* for 15 minutes. Extracellular proteins, associated with the outer membrane, were washed from the cell surface, using solutions of 9 M Urea, 1 M NaCl or 1% SDS, which were added to the pellets and incubated for 1 hour at room temperature. On completion, 50 mM Tris-HCl pH 7.7 was added to each wash solution in the volume ratio 9∶1. The resulted solutions of membrane-free proteins were applied to the polyacrylamide gel during zymography.

Briefly, 7 µl of 4×stock solution of lysis buffer (200 mM Tris-HCl, pH 6.8; 4% SDS; 0.01% bromophenol blue; 40% glycerol) were added to 20 µl of protein solutions. 7.5% polyacrylamide gel containing 0.2% CMC (sodium salt, medium viscosity, Sigma) was prepared and SDS-PAGE was performed without boiling the sample before application to the gel. After electrophoresis, gels were rinsed from SDS in water and incubated in 50 mM Tris-HCl pH 7.7 at 52°C for 1.5 h. Gels were stained using Congo Red solution as follows: 0.1% aqueous solution of Congo Red (Sigma) for 30 minutes (staining), 1 M NaCl 2 times per 30 minutes (de-staining) and 5% acetic acid (fixing). White bands reflecting active endoglucanases were observed on the blue background.

## Results and Discussion

### General Features of the *M. roseus* Genome

*Melioribacter roseus* has a single circular chromosome of 3,300,414 bp without any extrachromosomal elements. The GC content of the genome is 41%, - an average between the values characteristic for *Bacteroidetes* and *Chlorobi* phyla [11]. A single 16S-23S-5S rRNA operon and 45 tRNA genes coding for all of the 20 amino acids were identified. The chromosome displays two clear GC skew transitions that likely correspond to the DNA replication origin and terminus; the likely origin of replication was identified in the 303 bp region between the genes *dnaA* and *dnaN* (Fig. S1).

Annotation of the genome sequence revealed 2,838 potential protein-coding genes with an average length of 1,068 bp, covering 92% of the genome, of which 2133 (75%) can be functionally assigned. The functions of the remaining 705 genes could not be predicted from the amino acid sequences; of them, 272 genes are unique to *M. roseus* with no significant similarity to any known sequences.

The genome contains three CRISPR loci contained 16, 20 and 21 repeats units. The first CRISPR locus is linked to an operon, containing cluster of CRISPR-associated (*cas*) genes. In particular this operon includes *cas1, cas2*, and *cas3* genes. The latter gene encodes a large protein with helicase and DNAse activities that specify the type I CRISPR-Cas system [27]. The second CRISPR locus is associated with extra copies of genes coding for Cas1 and Cas2 proteins, while the third CRISPR is not linked to *cas* genes. CRISPR elements are widespread in bacterial genomes, and are considered an ancient antiviral defence system in the microbial world. The spacer regions are supposed to be derived from extrachromosomal elements like viruses [28], but none of the *M. roseus* spacers matches known plasmids or phages. Regarding the *cas* genes, the first CRISPR system of *M. roseus* is related to those found in *Firmicutes*, particularly in thermophilic cellulosolytic bacteria of the genus *Caldicellulosiruptor*, while *cas* genes of the second locus are closely related to those from *Bacteriodetes*. Therefore, one or both of the *M. roseus* CRISPR/Cas systems may have been independently horizontally acquired and followed by the loss of duplicated *cas* genes.

### Phylogenetic Placement of *Ignavibacteriae* as Revealed by Whole Genome Analysis

The first cultivated representative of *Ignavibacteriae*, *I. album*, was originally placed within the phylum *Chlorobi*, and was classified as the novel class *Ignavibacteria* [14]. Subsequent phylogenetic analysis of *M. roseus*, based on rRNA and RecA phylogenies suggested that *M. roseus* and *I. album* may be classified into the novel phylum *Ignavibacteriae* within Bacteroidetes/Chlorobi group [15].

**Figure 1:**
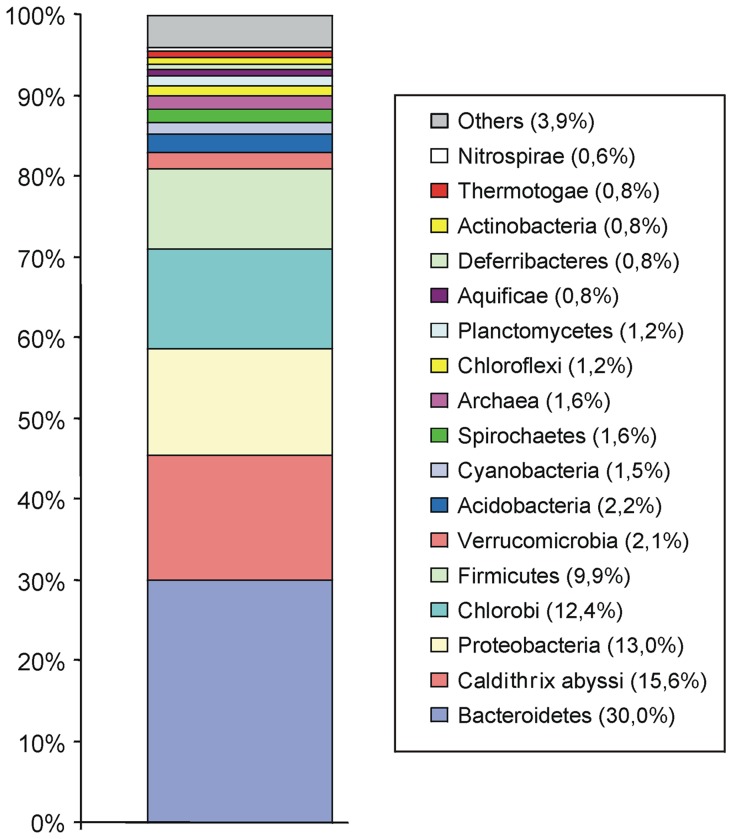
The taxonomic distribution analysis of *M. roseus* proteome.

To help further resolve this phylogenetic placement, a taxonomic distribution analysis was performed [29] by comparing each predicted protein in the *M. roseus* genome against the NCBI non-redundant database (excluding proteins from *I. album*). For each *M. roseus* protein with a match, the taxonomic identity of its top BLASTP hit was determined and the total number of proteins in *M. roseus* that have their closest match to microbes belonging to a given phylum was counted, as shown in Figure 1. If *M. roseus* is closely related to bacteria in a particular phylum, it is expected that a majority of *M. roseus* proteins would have close matches to proteins in bacteria belonging to that phylum. However, this study found that *M. roseus* proteins best matches ones in several phylum-level bacterial lineages, primarily *Bacteriodetes* (30%), *Proteobacteria* (13%), *Chlorobi* (12%), *Firmicutes* (10%), and *Caldithrix abyssi* (16%). The latter bacterium is a moderately thermophilic anaerobe isolated form a deep-sea hydrothermal chimney, and on 16S rRNA phylogenetic trees this isolate is very distant from any recognised bacterial phyla [30]. These data support, on a whole-genome scale, that *M. roseus* belongs to a separate phylum. Comparative genome analysis confirmed the close relationship of *M. roseus* and *I. album*, since the *I. album* proteome accounted for 56% of the best BLASTP hits of *M. roseus* proteins.

**Figure 2:**
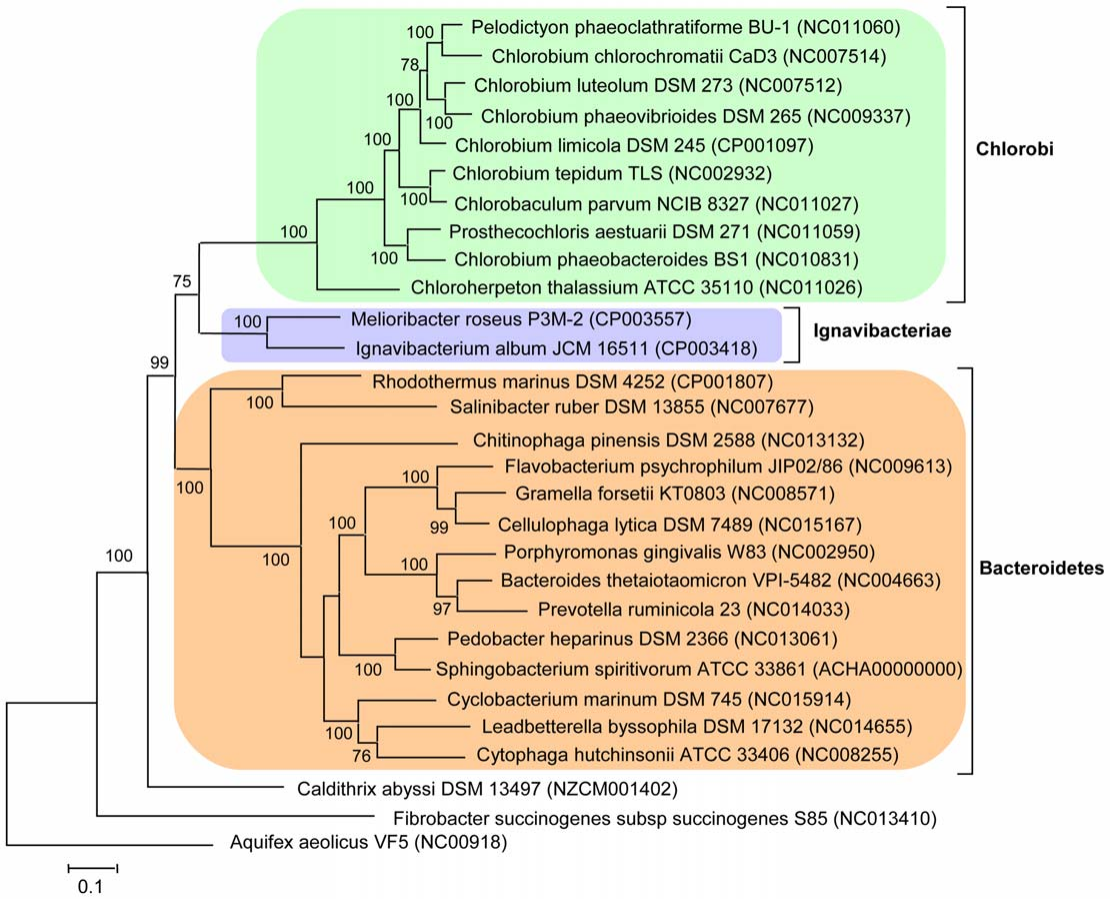
Maximum likelihood tree based on the concatenation of 39 ribosomal proteins. The use of the neighbour-joining method resulted in a similar tree topology. The ribosomal proteins from *Aquifex aeolicus* VF5 were used as an outgroup. Numbers at nodes represent bootstrap values (100 replications of the original dataset), only numbers above 50% are shown. The scale bar represents the average number of substitutions per site.

In order to resolve the phylogenetic position of *Ignavibacteriae*, genomic information of *M. roseus* and *I. album* was used alongside those from other complete bacterial genomes to perform phylogenetic analysis based on concatenated ribosomal proteins (Fig. 2). The results are consistent with rRNA phylogenies [15] and show that *Ignavibacteriae* forms a separate lineage branching close to the root of *Chlorobi* phylum after the divergence of *Bacteriodetes* and *Chlorobi.* On the phylogenetic tree of concatenated ribosomal proteins (Fig. 2 and Fig. S2 for a larger dataset), *C. abyssi* appeared to be affiliated with the group comprising *Fibrobacteres*, *Bacteriodetes* and *Chlorobi,* which may explain above mentioned high fraction of *C. asyssi* among the best hits for *M. roseus* proteins.

Some other particular features support the separation of *Ignavibacteria* from *Chlorobi*. The most notable is an absence in the *M. roseus* genome of genes involved in photosynthesis, including *fmoA* encoding the Fenna-Matthews-Olson antenna protein [31], a frequently used phylogenetic marker of *Chlorobi*. The *M. roseus* genome did not include *nif* genes, encoding MoFe-nitrogenase and its assembly proteins, which are highly conserved and universally present in all *Chlorobi*, except for the aerobic photoheterotroph *Candidatus* ‘*Thermochlorobacter aerophilum*’, being the deepest lineage within *Chlorobi* [32]. The absence of genes for photosynthetic machinery and nitrogen fixation correlates with facultatively anaerobic heterotrophic lifestyle of *M. roseus*. The ability of *M.* *roseus* to grow aerobically correlates with the presence of genes encoding superoxide dismutase and catalase, which are involved in the detoxification of the by-products of oxygen respiration. Another notable feature of *M. roseus* is its flagellar motility consistent with the presence of genes encoding flagellation proteins, absent in *Chlorobi* [15].

### Polysaccharide Degradation and Utilisation

*M. roseus* was isolated from a microbial mat, covering the wooden surface of a chute, under the flow of hot water (46°C) coming out of a deep oil exploring well [15]. One of the hallmarks of *M. roseus* is its ability to efficiently hydrolyse different plant polysaccharides, including microcrystalline cellulose, carboxymethyl cellulose, starch, dextrin, dextran, lichenan, agarose and xylan [15]. A number of low molecular weight carbohydrate substrates (glucose, mannose, rhamnose, arabinose, cellobiose, xylose, trehalose, ribose, maltose, fructose, lactose, and glycogen) can also support its growth [15]. These characteristics indicate that the metabolic versatility of *M. roseus* is similar to that of *Bacteroidetes*, which are well-known as active carbohydrate utilisers. This is reflected in *M. roseus* genome encoding extensive set of extracellular and intracellular enzymes responsible for polysaccharide degradation.

**Figure 3:**
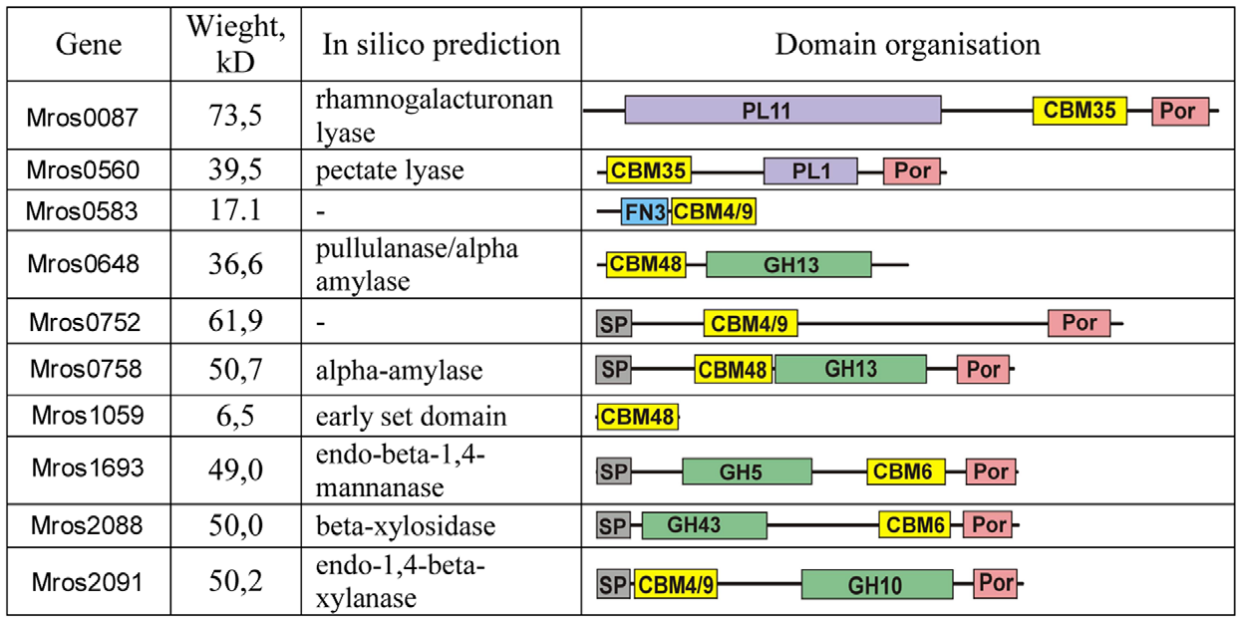
Carbohydrate-active enzymes containing CBM domains. Abbreviations: PL, polysaccharide lyase; CBM, carbohydrate binding module; Por, Por secretion system C-terminal sorting domain; FN3, fibrinonectin type 3 domain; GH, glycoside hydrolase; SP, signal peptide.

A search for carbohydrate-active enzymes (CAZy) [33] revealed more than 100 genes that were classified into 31 different families of glycoside hydrolases (GHs), carbohydrate esterases (CEs), and polysaccharide lyases (PLs) (Tables S1). A number of predicted carbohydrate-active enzymes contain carbohydrate binding modules (CBMs), facilitating their interaction with polysaccharide substrates (Fig. 3). Most of the CBM-containing enzymes also possess a C-terminal Por sorting domain, which is associated with the sorting of proteins to the outer membrane and the covalent modification in *Fibrobacteres* and *Bacteroidetes*. In addition to glycoside hydrolases, the *M. roseus* genome encodes several dozen glycoside transferases that could be involved in the degradation and synthesis of polysaccharides. Many of the *M. roseus* CAZymes are predicted to have signal peptides, indicating that these enzymes are targeted outside of the cytoplasm.

**Figure 4:**
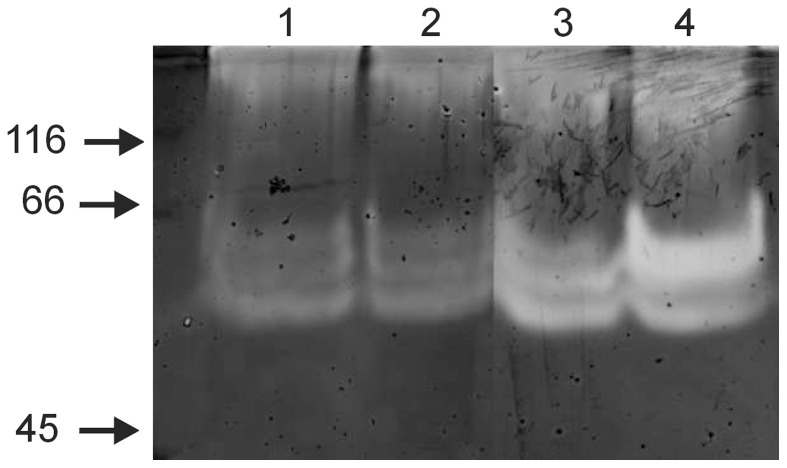
Zymographic analysis of hydrolytic activities against carboxymethyl cellulose. 1 - Proteins washed from the surface of aerobically grown cells with 1 M NaCl; 2 - Proteins washed from the surface of aerobically grown cells with 1% SDS; 3 - Proteins washed from the surface of anaerobically grown cells with 9 M urea; 4 - Proteins washed from the surface of aerobically grown cells with 9 M urea. Positions of molecular weight markers (sizes are shown in kD) are indicated by arrows. Note that the intracellular protein fraction and culture supernatant showed no activity against CMC on zymograms.

One of the most distinct features of *M. roseus* is its ability to digest microcrystalline and carboxymethyl cellulose in both aerobic and anaerobic conditions. Cellulose utilisation by the most well-studied microorganisms involves the activities of multiple enzymes, including endo-β-1,4-glucanases, cellobiohydrolases (also called exo-β-1,4-glucanases), and β-glucosidases, which act synergistically to convert crystalline cellulose to glucose [34]. Examination of the *M. roseus* genome identified many genes encoding possible cellulolytic enzymes (Table S1). These include possible endo-beta-1,4-glucanases belonging to glycoside hydrolase families GH5 (Mros0504, 0753, 0960, 2625) and GH9 (Mros0757, 2241, 2626, 2837). The calculated molecular weights of putative endoglucanases (Table 1) are in good agreement with the positions of bands detected by zymography of the cell-bound protein fraction with carboxymethyl cellulose. The results shown in Fig. 4 indicate the presence of two or three enzymes with molecular weights between 55 and 65 kD: Mros0757 and Mros2241 being the likely candidates. Notably, none of the putative cellulase genes contain CBMs of families 2 or 3, which are associated with binding to crystaline cellulose, and only a single GH5 family enzyme (Mros1693), which is predicted to encode endo-beta-1,4-mannanase, contains a CBM6 domain enabling binding to cello-oligosaccharides, laminarin, xylo-oligosaccharides and beta-1,4, beta-1,3 mixed glucans [35]. None of the cellulase genes contain dockerin and scaffoldin domains, confirming the absence of cellulosomal structures in *M. roseus*.

**Table 1:** Endoglucanases predicted from the *M. roseus* genome.

Gene	GH family	Pfam domains	Signal peptide	Calculated weight (kD)
Mros0504	5	GH5	+	41.6
Mros0753	5	GH5	+	38.8
Mros0757	9	CelD_N/GH9	+	62.4
Mros0960	5	GH5	+	96.6
Mros2241	9	CelD_N/GH9	+	58
Mros2625	5	GH5	−	43.2
Mros2626	9	CelD_N/GH9/GH9	−	84.5
Mros2837	9	CelD_N/GH9	+	96.3

Genes encoding proteins with a similarity to known cellobiohydrolases, which are typically members of the GH6 or GH48 families in bacteria [36]; [37], were not detected. However, it is known that several cellobiohydrolases belong to families GH5 and GH9, which were found in the *M. roseus* genome. Beta-glucosidases belonging to the GH3 (Mros0754 and others) and GH1 (Mros0638) families, as well as cellobiose phosphorylases of the GH94 family (Mros1576, 1712 etc.), were also found in the genome.

Most aerobic microorganisms secrete a set of cellulases, containing carbohydrate- binding modules that serve to bind the catalytic domains to insoluble substrates, outside the cell (free cellulase mechanism) while anaerobic microorganisms produce large multi-enzyme complexes on their outer surface (cellulosomes), although not many organisms have been found to use it so far [38]. Several microorganisms possess their own mechanism, like aerobic *Cytophaga hutchinsonii* and anaerobic *Fibrobacter succinogenes*, which do not produce free cellulases and cellulosomes [39]. A possible mechanism of *C. hutchinsonii* cellulose degradation, proposed by Wilson [39], involves the action of the protein complex, located on the surface of the outer membrane, which is able to remove individual cellulose molecules from cellulose fibres and transport them through the outer membrane into the periplasmic space, where they would be cleaved by endoglucanases. Extracellular endocellulases might function by producing cellulose ends for binding to the outer membrane component of the cellulose-degrading machinery. This mechanism resembles one used by *Bacteroidetes* (*sus* operon) for starch degradation [40]. A similar mechanism was proposed for *F. succinogenes*; however it uses different outer membrane proteins for binding, removing and transporting cellulose molecules [39]; [41]. Taking into account the fact that the majority of *M. roseus* endoglucanases lacking CBMs have homologs in *C. hutchinsonii* and *F. succinogenes*, while the cellulolytic activity was associated with the cell surface (Fig. 4), it is proposed that the cellulolytic mechanism of *M. roseus* resembles that of *C. hutchinsonii* and *F. succinogenes*.

*M. roseus* possesses genes encoding a wide range of glycoside hydrolases that are active against various polysaccharides used by this organism for growth. The genome encodes GHs for the hydrolysis of xylan (Mros2090, 2091 etc.), mannanan (Mros0511, 1693), lichenan (Mros0976), and agarose (Mros0970). Some of the hemicellulases contain CBM6 domains and CBM4/CBM9 domains binding to amorphous [42] and crystalline [43] cellulose, among other substrates. In particular, GH10 hydrolase Mros2091, containing a CBM4/CBM9 domain and a signal peptide enabling its export from the cell, may be active towards xylan. This gene is a part of a cluster encoding several enzymes involved into xylan metabolism: beta-xylosidases (Mros2087–2088), glucuronoarabinoxylan endo-1,4-beta-xylanase (Mros2090), and alpha-N-arabinofuranosidase (Mros2093). All of the enzymes, except for Mros2087, contain signal peptides indicating their extracellular operation, while Mros2088 also contains a CBM6 domain.

The utilisation of starch may be enabled by several amylases that are sporadically distributed in the genome (Mros0648, 1330, 1936 etc.), as well as by a *sus* operon (Mros0764-0758), which is common among *Bacteroidetes* and has been well characterised for *Bacteroides thetaiotaomicron* [40]. One enzyme, alpha-amylase of GH13 family (Mros0758), was predicted to be secreted out of the cell and carry a CMB48 domain. This CBM is found in a range of enzymes that act on branched substrates: isoamylase, pullulanase and branching enzyme, suggesting that Mros0758 may have broad substrate specificity. Two other enzymes, the GH13 family alpha-amylase (Mros0762) and the GH31 family alpha-glucosidase (Mros0763), probably, act in the periplasm for the complete hydrolysis of maltooligosaccharides and maltose. This operon also encodes the outer membrane proteins SusC, SusD and SusE (Mros0761-0759) involved in binding starch on the cell surface and the transport of maltose and maltoolisaccharides across the outer and inner membranes. Another interesting point is that three genes of enzymes possibly involved in cellulose hydrolysis (endo-beta-1,4-glucanases Mros0753 and Mros0757, and beta-glucosidase Mros0754) are located immediately downstream of the *sus* operon. Thus, it is possible that *M. roseus* may use SusCDE also for cellulose binding and the transport of cellooligosaccarides and cellobiose into the cell. A similar hypothesis was made for the cellulose utilisation mechanism of *Cytophaga hutchinsonii* [44], which does not grow on starch.

Several polysaccharide lyases and carbohydrate esterases were identified, and most of them were predicted to act as pectate lyases (Mros0560 etc.) and pectin methylesterases (Mros0879 and Mros2353). Some enzymes contained a signal peptide and carbohydrate binding modules, indicating their extracellular localisation, although the growth of *M. roseus* on pectin was not confirmed [15]. The possible explanations might be that (1) *M.* *roseus* use pectin esterases to release cellulose and hemicelluloses for their further hydrolysis, as it is known for a highly-specialised cellulolytic bacterium, *Fibrobacter succinogenes,* [41] which hydrolyses a wide range of polysaccharides while metabolising only cellulose, or/and (2) the synthesis of pectin esterases might be induced by another component, like galacturonic acid or galactose, as shown for *Neurospora crassa* [45]. One gene encoding PL6, a putative alginate lyase, was also found (Mros1361). Another explanation could be simply that *M. roseus* is able to grow on particular types of pectin other then one tested in [15].

The genome of *M. roseus* contains two genes (Mros0583 and Mros0752) that are predicted to encode proteins with CBM4/9 domains, but which have no apparent catalytic activity. The function of these proteins is unclear. Notably, the product of gene Mros0752 contains a signal peptide, suggesting the extracellular operation of this protein.

The *M. roseus* genome encodes several dozen glycoside transferases, which are involved in the synthesis of intra- and extracellular polysaccharides. Two putative GT5 family glycogen synthases (Mros0053, Mros1119) are necessary for the synthesis of intracellular glycogen and/or extracellular alpha-glucan. GT2 enzymes (Mros1224, 1401, 1750 etc.) might be involved in the formation of various polysaccharides, including cellulose, chitin, beta-mannan and alginate. Several GT4 transferases (Mros0374, 0714, 1314, 1315 etc.) might perform the synthesis of sucrose or trehalose. In some cases, GTs retaining catalytic mechanisms theoretically may perform the opposite reaction, hydrolysis of oligosaccharides.

The genome of *M. roseus* encodes more than a hundred transporters of different families and specificities. Among them, carbohydrate transport might occur via MFS (2.A.1 according to Transporter Classification Database [46]) transporters (Mros0115, 0508, 2077, 2102 etc.), SSS (2.A.21) transporters (Mros0031, 0509 etc.) and TonB (2.C.1) transporters (Mros0761 etc.).

Analysis of the *M. roseus* genome reveals the presence of genes encoding type IV pili genes (*pilT, pilQ, pilB, pilC*). Although a complete set of genes necessary to produce pili is probably not present since genes *pilA* and *pilD* were not found, these pilin proteins may be used by *M. roseus* to adhere to cellulose in a manner similar to how *Escherichia coli* and other gram negative bacteria adhere to solid substrates [47]. The reliance of *M. roseus* on membrane-bound adherence proteins may also be correlated to its protein secretion systems. *M. roseus* appears to encode the Sec-dependent and type II secretion pathways. In other bacteria such as the plant pathogen *Xanthomonas campestris*, the Sec-dependent and type II systems are used to secrete hydrolytic enzymes, like cellulases, chitinases, and pectate lyases, in order to facilitate its pathogenic lifestyle [48]. Given that *M. roseus* requires adherence to cellulose for efficient hydrolysis, this pathway may play a role in cell adherence.

### Central Metabolism

**Figure 5:**
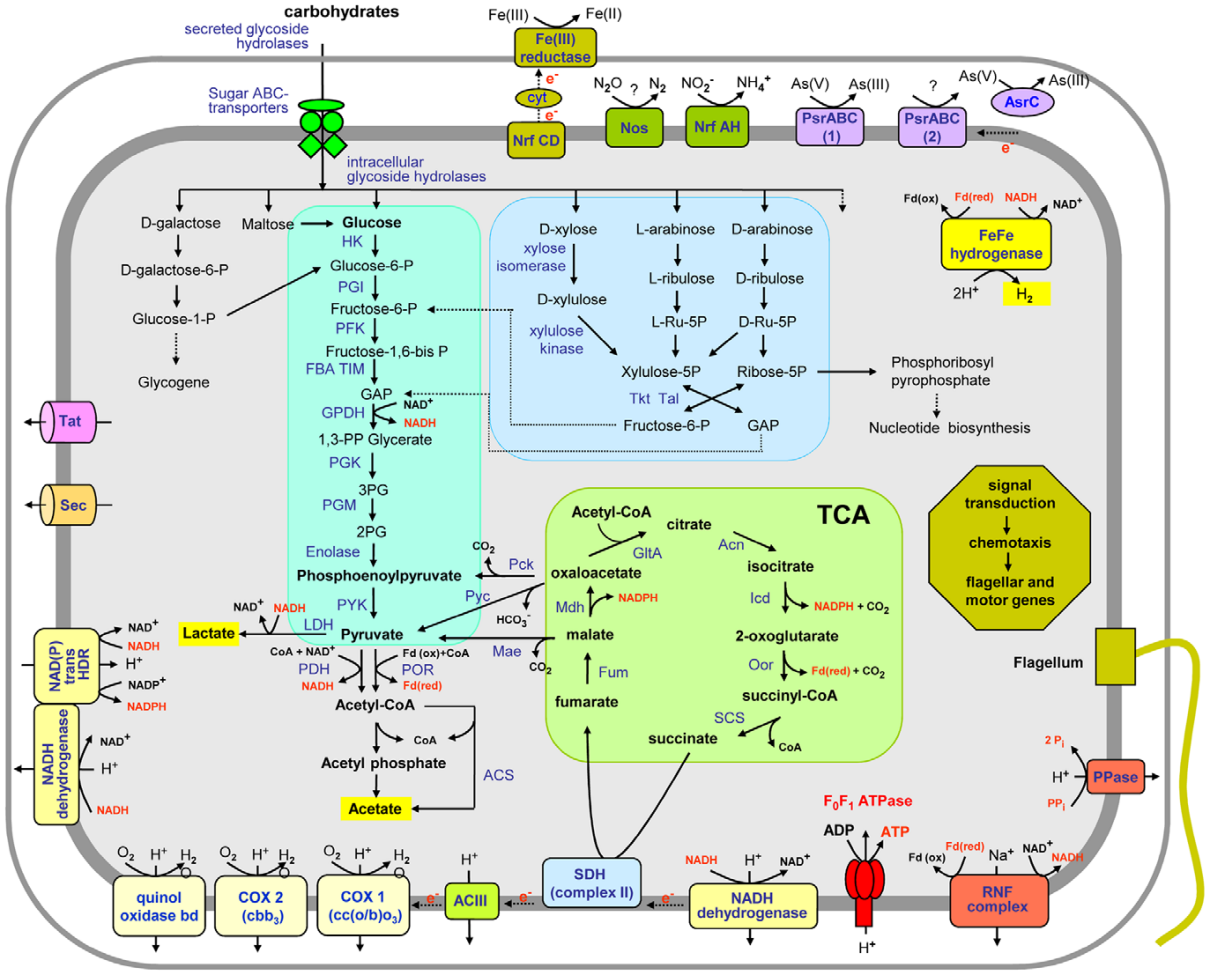
An overview of the metabolism of *M. roseus*. Enzymes and proteins identified in the genome are in blue, energy-rich intermediate compounds are in red. Enzyme abbreviations: HK, hexokinase; PGI, phosphoglucose isomerase; PFK, phosphofructokinase; FBA, fructose-bisphosphate aldolase; TIM, triose phosphate isomerase; GPDH, glyceraldehyde 3-phosphate dehydrogenase; PGK, phosphoglycerate kinase; PGM, phosphoglycerate mutase; PYK, pyruvate kinase; LDH, lactate dehydrogenase; PDH, pyruvate dehydrogenase; POR, pyruvate ferredoxin oxidoreductase; ACS, acetyl-CoA synthetase; Pyc, pyruvate carboxylase; Mae, malic enzyme; Pck, phosphoenolpyruvate carboxykinase; GltA, citrate synthase; Acn, aconitase; Icd, isocitrate dehydrogenase; Oor, 2-oxoglutarate ferredoxin oxidoreductase; Fum, fumarase; Mdh, malate dehydrogenase; SDH, succinate dehydrogenase; Tkt, transketolase; TalB, transaldolase; ACIII, alternative complex III; COX, cytochrome c oxidase; ArsC, arsenite reductase; NAD(P) trans HDR, NAD(P) transhydrogenase; Nos, nitrous oxide reductase; PPase, pyrophosphatase. Other abbreviations: GAP, glyceraldehyde-3-phosphate; 3-PG, 3-phosphoglycerate; 2-PG, 2-phosphoglycerate; L-Ru-5-P, L-ribulose-5-phosphate; D-Ru-5-P, D-ribulose-5-phosphate; Fdox/Fdred, ferredoxin, oxidized and reduced form; Cyt, cytochrome; Pi, phosphate; PPi, pyrophosphate; CoA, coenzyme A.

*M. roseus* did not grow photo- or chemoautotrophically and is able to ferment polysaccharides producing H_2_, acetate and CO_2_ as the main products of fermentation, although traces of lactate were also detected [15]. The genome of *M. roseus* contains a complete set of genes encoding enzymes of the Embden-Meyerhof (EM) pathway of glucose metabolism. Pathways for the utilisation of other sugars are linked to the EM pathway (Fig. 5). For example, xylose, a major constituent of hemicellulose, is metabolised by the action of a putative xylose isomerase (Mros2028) and a xylulose kinase (Mros2029), that enter into the non-oxidative branch of the pentose phosphate pathway (PPP). The non-oxidative branch of the PPP uses ribulose phosphate 3-epimerase (Mros2501), ribose-5-phosphate isomerase (Mros0016 and Mros0521), transketolase (Mros0693 and Mros2334), and transaldolase (Mros1726) to produce fructose-6-phosphate and glyceraldehyde-3-phosphate, entering into the EM pathway. Besides the metabolism of pentoses, the non-oxidative PPP allows the production of intermediates necessary for nucleic acid synthesis (Fig. 5).

The pyruvate generated in the EM pathway is oxidised to acetyl-CoA by pyruvate:feredoxin oxidoreductase producing reduced ferredoxin, or by the pyruvate dehydrogenase complex (Mros0859-857), while lactate dehydrogenase may account for the production of lactate. Acetyl-CoA is converted to acetate with the concomitant production of ATP either via a single-step reaction by an acetyl-CoA synthetase (Mros_1085 and Mros_2210) or a two-step reaction by phosphate acetyltransferase (Mros2846) and acetate kinase (Mros0094 and 1926). In the presence of external electron acceptors, acetyl-CoA may be completely oxidised via the TCA cycle, which is encoded by the *M. roseus* genome (Fig. 5). However, unlike all *Chlorobi*, including photoheterorophic species ‘*Ca*. T. aerophilum’ [32], *M. roseus* lacks the ATP-dependent citrate lyase gene that makes the reductive TCA cycle incomplete, unless this function is performed by another enzyme. The key enzymes of the Calvin-Benson-Bassham, 3-hydroxypropionate, and Wood–Ljungdahl CO_2_-fixing pathways were also not identified.

In the presence of oxygen or other external electron acceptors, *M. roseus* was capable of respiratory growth on acetate [15], which is a principal fermentation product in microbial mats [49]. Acetate can be activated to acetyl-CoA which then could be carboxylated by pyruvate:ferredoxin oxidoreductase to form pyruvate, or it could enter the oxidative TCA cycle.

Three hydrogenases of the [FeFe]-family are encoded by the *M.* *roseus* genome. The catalytic subunits (Mros0634, Mros2482 and Mros2488) contain the three characteristic sequence signatures of the active site, H-cluster [50]; [51], and additional N-terminal domain, which accommodate 4Fe-4S clusters. These hydrogenases are similar to heterotrimeric [FeFe] hydrogenase from *T. maritima* [52]. The catalytic subunits are parts of operons comprising two accessory subunits, encoded by genes Mros0635-636, Mros2481-2480, and Mros2487 (fused). The *T. maritima* enzyme oxidises NADH and ferredoxin simultaneously in a synergistic fashion to produce H_2_. It represents a class of bifurcating [FeFe] hydrogenases in which the exergonic oxidation of ferredoxin is used to drive the unfavourable oxidation of NADH to produce H_2_ [52]. All of the components of *M. roseus* hydrogenases contain no recognisable signal peptides or transmembrane helices, suggesting their operation in the cytoplasm. During the anaerobic fermentation of sugars by *M. roseus* in the EM pathway and the subsequent oxidation of pyruvate to acetate, both NADH and reduced ferredoxin are generated. Activity of the bifurcating [FeFe]-family hydrogenases in *M. roseus* will couple the oxidation of reduced ferredoxin and NADH to the reduction of protons to H_2_, which is detected as one of the fermentation products.

### Respiratory Electron Transfer Chains

The genome of *M. roseus* encodes all of the major components of the electron transfer chain necessary for energy generation via oxidative phosphorylation, namely, the proton-translocating NADH-dehydrogenase complexes, membrane-bound succinate dehydrogenase (complex II), isoprenoid quinones and the alternative complex III (ACIII) quinol oxidase. The resulting transmembrane proton gradient may be used for ATP generation by the encoded membrane F_0_F_1_-type ATP synthase.

The *M. roseus* genome contains two main clusters encoding most of the subunits of a type I NADH dehydrogenase, *ndhABCDHIJKLMN* (Mros2379-2369), and *ndhABCDEFJKLMN* (Mros0160-0155, 0138-0135, and 0132). The third locus comprises the genes *ndhGHI* (Mros2032-2034). The *ndh* genes from the first and the third clusters are closely related to those found in *Chlorobi* while close homologs of the subunits encoded by the second cluster were found in various bacterial lineages. Together, the three clusters encode a full set of the 14 *ndh* genes required for the assembly of NADH:quinone oxidoreductase. However, the absence of genes encoding the NuoEFG subunits that are known to form the dehydrogenase domain involved in NADH binding and oxidation [53] in the first cluster suggest the possibility that it encodes an alternative membrane-bound proton-transporting complex accepting electrons from donor besides NADH (e.g. reduced ferredoxin).

The first NDH cluster is linked to the genes Mros2380-2382, encoding membrane-bound nicotinamide nucleotide transhydrogenase. These enzymes couple the transfer of reducing equivalents between NAD(H) and NADP(H) to the inward translocation of protons across a membrane in the reaction NADH+NADP^+^+H^+^_out_ = NAD^+^+NADPH+H^+^_in_ [54]. The resulting NADPH can be used in biosynthesis reactions. Therefore, this enzyme consumes the proton electrochemical gradient generated by NADH dehydrogenase.

The succinate dehydrogenase complex II is encoded by a three-gene operon (Mros0459-457) and consists of an iron-sulfur subunit (Mros0457), a flavoprotein subunit (Mros0458) and a transmembrane cytochrome *b* subunit (Mros0459). The organisation of this complex is typical for *Bacteroidetes* and *Chlorobi*.

In most bacteria, complex III, which is also called cytochrome *bc_1_*, mediates electron transfer between complex I or II and complex IV by accepting electrons from reduced quinones and reducing cytochrome c. However, inspection of the *M. roseus* genome revealed no cytochrome *bc_1_* genes; instead the so-called alternative complex III (ACIII) is encoded. This complex, initially discovered in *Rhodothermus marinus* [55], is structurally different from the cytochrome *bc1* complexes, even though it performs the same function. As in some other organisms [56]; [57], in *M. roseus* the genes encoding ACIII (Mros0045-0040) and cytochrome *c* oxidase genes (Mros0039-0033) are clustered in the genome. The same genomic context has been reported for ACIII of *R. marinus*, which is able to directly interact with oxygen reductase, but not for ACIII transferring electrons to periplasmic acceptors [56]; [57]. Thus, it is likely that the ACIII complex is involved in aerobic rather than anaerobic respiratory processes. This assumption is further supported by the fact that a class I soluble c551/c552 cytochrome (Mros0033), encoded upstream of the cytochrome *c* oxidase, represents a common component of the aerobic respiratory electron transfer chains that shuttle electrons between complex III and terminal oxidases, suggesting that ACIII functions in respiration. The subunits of *R. marinus* ACIII can be divided in two groups according to the proposed function [57]: membrane attachment and quinone interaction modules (ActCDFG), and electron transfer modules (ActABE). The *actA* gene (Mros0045) codes for a cytochrome protein containing four haem C binding motifs. The catalytic subunits ActB and ActC are encoded by genes Mros0044 and Mros0043, respectively. The cytochrome C family protein coded by gene *actE* (Mros0040) contains a single CXXCH motif, and the conserved motif MPA present in other cytochromes [58]. The Mros0042 gene codes for a protein predicted to have three transmembrane helices and a single CXXCH motif in the C-terminal part, and it is thought that the encoded protein is a fusion between ActD and ActE. Finally, the *actF* gene (Mros0041) codes for another integral membrane protein, predicted to have nine transmembrane helices. A homolog of the *R. marinus* gene *actG* was not found in the *M.* *roseus* genome.

### Rnf Complex

Analysis of the *M. roseus* genome revealed an operon containing a set of six genes (*rnfCDGEAB*, Mros1482–1478, 1476) that are related to the potential membrane-bound electron transport complex (Rnf) found in various bacteria. Two other genes, Mros1477, encoding a putative lipoprotein, and Mros1475, encoding a protein with an unknown function, are apparently components of the *rnf* operon, which suggests that they may be responsible for related functions. The complex was first discovered in the *Rhodobacter capsulatus* [59] where it transports electrons from NADH to ferredoxin, which donates electrons to nitrogenase [60]. It was further proposed that the same complex runs in an opposite direction, acting as an energy-conserving ferredoxin:NAD+ oxidoreductase complex [61]; [62]. This complex catalyses the oxidation of reduced ferredoxin coupled with the reduction of NAD^+^, a process which is coupled with the electrogenic pumping of Na^+^ or H^+^ ions across the membrane out of the cell. The ion gradient can be used for ion (Na^+^/H^+^)-dependent symporters to take up sugars and amino acids into the cell, and/or by membrane ATP sythase for ATP generation. Thus, the Rnf complex represents a unique type of primary ion pump and an evolutionary mechanism of energy conservation. The proteins of the Rnf complex of *M. roseus*, as well as the gene order of the operon, are most similar to those found in *Firmicutes*, while another variation *rnfBCDGEA* is found in *Bacteroidetes* and *Chlorobi* [62].

### Terminal Oxidoreductases of Aerobic and Anaerobic Respiratory Pathways

*M. roseus* prefers aerobic conditions as indicated by the fact that at optimal conditions the doubling time was 30 minutes for aerobic growth and 2.5 hours for anaerobic growth [15]. Terminal oxygen reductases in *M. roseus* are represented by two proton translocating cytochrome *c* oxidases and a quinol oxidase *bd* complex. The quinol oxidase *bd* complex is encoded by the gene cluster Mros0842–0843. The first gene, Mros0843, or *cydA*, encodes subunit I, which is predicted to comprise nine transmembrane helices, and two *b*-type haems. Mros0842, or *cytB*, encodes the second subunit comprising eight transmembrane regions and one *d*-type haem. Both proteins are most similar to subunits of the *bd* complex of the delta proteobacteria of the order *Myxococcales*, and its homologs are also present in phototrophic *Chlorobi*.

The first cytochrome *c* oxidase-encoding cluster is composed of seven genes (Mros0039-0033), which are organised as a putative operon. The Mros0038 and Mros0035 genes encode the catalytic subunits CoxI and CoxII, respectively, while Mros0037 and Mros0038 encode the transmembrane subunits CoxIII and CoxIV, respectively. Protein sequences and gene order are similar to that of delta-proteobacteria of the orders *Desulfovibriales* and *Desulfuromonadales*, suggesting that this cluster was laterally transferred between *Ignavibacteriae* and a common ancestor of *Desulfovibriales* and *Desulfuromonadales* [15].

Another putative membrane-bound cytochrome *c* oxidase complex is encoded by the eight gene operon Mros1511–1518. This cluster encodes the *cbb3*-type cytochrome *c* oxidase, composed of fused subunits I and II (Mros1513), III (Mros1515) and IV (Mros1514). Other genes in this cluster encode cytochrome *c* oxidase accessory proteins and a subunit of the E1–E2 ATPase family. *M. roseus cbb3*-type cytochrome *c* oxidase clearly belongs to type C [63], found in *Bacteroidetes* and *Chlorobi*. The *cbb3*-type oxidases are widespread among bacteria and usually have a very high affinity for O_2_ [64]. For example, the human pathogen *Campylobacter jejuni* has a branched aerobic electron transport chain, with a *cbb3*-type oxidase of high O_2_ affinity (Km = 40 nM) and a quinol oxidase of low (Km = 800 nM) O_2_ affinity. The latter complex was mainly expressed at high O_2_ conditions, while *cbb3*-type oxidase was significantly induced in a low O_2_ environment [65]. Similarly, in *Rhodobacter capsulatus,* the activity of *cbb3*-type oxidase is high under microaerobic conditions, but low under fully aerobic or under fully anaerobic growth conditions [66]. Enzymes closely related to *M. roseus cbb3*-type cytochrome *c* oxidase were found in aerobic and microaerophilic *Bacteroidetes* bacteria, for example in *Cytophaga hutchinsonii*. Therefore, this oxidase may allow respiration of *M. roseus* under microaerophilic and even aerobic conditions.

Under anaerobic conditions *M. roseus* is able to grow by fermentation or anaerobic respiration. Fe(III), arsenate and nitrite may be used as electron acceptors, while elemental sulfur, thiosulfate, sulfite and nitrate did not stimulate growth [15]. Terminal reductases of anaerobic respiratory pathways are represented in the *M. roseus* genome by the nitrite-reductase complex, membrane-bound oxidoreductase complexes of the Complex Iron-Sulfur Molybdopterine (CISM) family and multi-haem *c*-type cytochromes.

Putative dissimilatory nitrite-reductase complex of NrfAH-type consists of the tetrahaem cytochrome c electron transfer subunit NrfH (Mros1099) and a catalytic pentahaem *c* type cytochrome subunit NrfA (Mros1098). The Mros1100 gene, encoding a putative electron transfer protein with five transmembrane helices, is located in the same operon.

The gene cluster predicted to encode cytochrome *c* nitrous oxide (N_2_O) reductase is located close to the *nrfHA* gene. This cluster comprises genes encoding the catalytic subunit NosZ (Mros1104), accessory proteins NosL (Mros1105), NosD (Mros1106), NosF (Mros1107) and NosY (Mros1108), cytochrome C (Mros1103), and a putative iron-sulfur protein (Mros1109). Transcription of this operon may be controlled by the BadM/Rrf2 family transcriptional regulator encoded by the Mros1101 gene. The actual physiological role of the predicted nitrous oxide reductase in *M. roseus* remains to be investigated since the ability of the bacterium to reduce N_2_O was not reported.

The genome analysis revealed two gene clusters encoding putative membrane bound molybdopterin oxidoreductases of the Psr/Phs family [reviewed in 67]. These membrane-bound complexes typically consist of three subunits: a catalytic subunit with molybdopterin (PsrA/PhsA), an electron transfer subunit with the [Fe–S] cluster (PsrB/PhsB), and a membrane anchor subunit, participating in the transfer of electrons from the quinone pool (PsrC/PhsC). The first operon, Mros1774–1776 (*psrABC*) encodes a typical three-subunit Psr/Psh oxidoreductase and also contains a putative MerR family transcriptional regulator gene (Mros1773). The subunits of this oxidoreductase are similar to the respective components of thiosulfate-, polysulfide-, nitrate-, and arsenate-reductases, as well as other related proteins. The catalytic subunit Mros1076 of the second oxidoreductase (Mros1074–1076) shares similarity with the respective component of tetrathionate reductases, which are enzymes responsible for the anaerobic reduction of tetrathionate to thiosulfate. The ability to respire tetrathionate is the characteristic of certain genera of *Enterobacteriaceae* [68], but it was not tested for *M. roseus*. Taking into account that the specificity of molybdopterin oxidoreductases of the Psr/Phs family cannot be reliably predicted from amino acid sequences, it is possible that the encoded complexes reduce arsenate and/or another yet unknown electron acceptor(s).

The NrfCD cluster encodes two subunits of the CISM family oxidoreductase: the NrfC-like 4Fe-4S ferredoxin iron-sulfur protein Mros1572 and the NrfD-like transmembrane protein (Mros1571). Such complexes lacking molybdopterine-binding catalytic subunits are known to serve as multifunctional quinol:electron acceptor reductases that are able to donate electrons to periplasmic multihaem *c*-type cytochromes [67]. This electron transfer chain may terminate at the hypothetical Fe(III) reductase linked to the outer membrane [15].

Analysis of the *M. roseus* genome revealed an additional site of arsenate reduction. This locus contains a four-gene arsenic resistance operon (Mros1066-1063) with a transcription regulatory factor of the ArsR family (Mros1067). The structural proteins of the Ars operon include a permease protein (Mros1066), a soluble arsenate reductase ArsC (Mros1065) of the low-molecular weight protein-tyrosine phosphatase family (pfam01451), a small redox-active disulfide protein (Mros1064), and a membrane-bound arsenite efflux pump (Mros1063). This arsenate reductase is predicted to be localised in the periplasmic space where it reduces arsenate to arsenite, which may be more toxic but is more easily exported from the cell [69]. This periplasmic enzyme complex serves a detoxifying function and seems not to be involved in the generation of a transmembrane ion gradient, but still may serve as an electron sink.

### Conclusions

Until recently, the bacteria of the *Chlorobi* phylum were considered a homogeneous group of obligately anaerobic photolitothrophs using reduced sulfur compounds and hydrogen as electron donors for photosynthesis. However, the discoveries of new lineages more distantly related to known *Chlorobi* extended our view of the metabolic diversity of *Chlorobi* and contributed to the understanding of the evolution of this group. ‘*Candidatus Thermochlorobacter aerophilum*’, identified in microbial mats of the hot springs at the Yellowstone National Park and characterised through metagenomic and metatranscriptomic approaches, appeared to be an aerobic photoheterotroph that cannot oxidise sulfur compounds, autotrophically fixes CO_2_ in the reverse TCA and does not contain nitrogenase machinery [32]. Similar environments were used to isolate two organisms even more distantly related to *Chlorobi: I. album* [14] and *M. roseus* [15]. Both cultivation and genome data show that *M. roseus* is a facultatively anaerobic heterotroph equipped with a diverse set of hydrolytic enzymes. Under anaerobic conditions it can grow either by fermentation or by anaerobic respiration in the presence of external electron acceptors. In the absence of such acceptors, *M.* *roseus* can ferment sugars in the EM pathway following the conversion of pyruvate to acetate. The reduced products that are generated, NADH and reduced ferredoxin, are reoxidised by bifurcating [FeFe]-family hydrogenases with a concomitant reduction of protons to H_2_. The transmembrane ion gradient, required for cation-dependent sugar symporters to transport sugars and aminoacids into the cell, and for membrane ATP sythase for ATP generation, can still be generated by the activity of the Rnf ion pump and vacuolar-type H(+)-translocating pyrophosphatase (Mros2367). In the presence of external electron acceptors, *M. roseus* can grow by aerobic or anaerobic respiration. Several membrane-bound oxidoreductases allow the complete oxidation of organic substances via the TCA cycle with the generation of a transmembrane proton gradient used by ATP synthase for the generation of ATP. The ability of *M. roseus* to grow under a wide range of oxygen concentrations up to aerobic conditions distinguishes it from strictly anaerobic phototrophic *Chlorobi*, and could be attributed to the presence of three different terminal oxidases: two different cytochrome *c* oxidases and a quinol *bd* oxidase. It is possible that these oxidases have different O_2_ affinities, thus being employed under different growth conditions. Transcription analysis should clarify the role of each enzyme complex.

Overall, the metabolic characteristics of *M. roseus* are consistent with the basal branching point of *Ignavibacteriae* on the phylogenetic tree of *Chlorobi* (Fig. 1) since they lack all of the characteristic features of the photoautotrophic *Chlorobi* and resemble heterotrophic free-living *Bacteriodetes*. Photosynthetic machinery appeared to be acquired first (*Ca.* ‘*C. thermophilum*’) followed by metabolic and environmental specialisation, - a shift to an obligate photoautotrophy linked to sulfide oxidation, the acquisition of nitrogen fixation machinery, the loss of flagellar motility and moving into strictly anaerobic, poorly illuminated ecological niches.

## Supporting Information

Figure S1 **A GC skew analysis of the** ***M. roseus*** **genome showing the major peaks where nucleotide compositional deviations occur.** In the bottom part of the figure the nucleotide sequence of predicted *oriC* region is shown. Open rectangles show the *dnaA* and *dnaN* genes flanking the *ori* site with arrows indicating the direction of transcription. Arrows show positions of the putative DnaA binding sites, predicted consensus sequence of the DnaA box is shown above.
(DOC)

Figure S2 **Maximum likelihood tree based on the concatenation of 39 ribosomal proteins.** The ribosomal proteins from *Aquifex aeolicus* VF5 were used as an outgroup. Numbers at nodes represent bootstrap values (100 replications of the original dataset). The scale bar represents the average number of substitutions per site. The following species of Proteobacteria and Firmicutes were used to construct the tree: *Alkalilimnicola ehrlichii* MLHE-1 (NC_008340), *Anaerococcus prevotii* DSM 20548 (NC_013171), *Anaeromyxobacter dehalogenans* 2CP-C (NC_007760), *Aromatoleum aromaticum* EbN1 (NC_006513), *Bacillus weihenstephanensis* KBAB4 (NC_010184), *Bartonella quintana* str. Toulouse (NC_005955), *Bordetella pertussis* Tohama I (NC_002929), *Bradyrhizobium japonicum* USDA 110 (NC_004463), *Caldicellulosiruptor bescii* DSM 6725 (NC_012034), *Campylobacter jejuni* subsp. jejuni NCTC 11168 (NC_002163), *Desulfitobacterium hafniense* Y51 (NC_007907), *Desulfohalobium retbaense* DSM 5692 (NC_013223), *Desulfomicrobium baculatum* DSM 4028 (NC_013173), *Dichelobacter nodosus* VCS1703A (NC_009446), *Erythrobacter litoralis* HTCC2594 (NC_007722), *Ferrimonas balearica* DSM 9799 (NC_014541), *Haliangium ochraceum* DSM 14365 (NC_013440), *Halothermothrix orenii* H 168 (NC_011899), *Halothiobacillus neapolitanus* c2 (NC_013422), *Helicobacter pylori* 26695 (NC_000915), *Herminiimonas arsenicoxydans* (NC_009138), *Listeria innocua* Clip11262 (NC_003212), *Magnetospirillum magneticum* AMB-1 (NC_007626), *Mahella australiensis* 50-1 BON (NC_015520), *Nautilia profundicola* AmH (NC_012115), *Neisseria meningitidis* MC58 (NC_003112), *Oenococcus oeni* PSU-1 (NC_008528), *Staphylococcus aureus* subsp. aureus N315 (NC_002745), *Thermoanaerobacter italicus* Ab9 (NC_013921), *Veillonella parvula* DSM 2008 (NC_013520).
(DOC)

Table S1 Carbohydrate-active enzymes encoded by the *M. roseus* genome.
(DOC)
