# Supplementary material for: Genomic Analysis of Melioribacter roseus, Facultatively Anaerobic Organotrophic Bacterium Representing a Novel Deep Lineage within Bacteriodetes/Chlorobi Group
Source: PLoS One. 2013 Jan 2;8(1):e53047. doi: 10.1371/journal.pone.0053047 (PMC3534657; doi:10.1371/journal.pone.0053047)
Supplement: Figure S1 — A GC skew analysis of the M. roseus genome showing the major peaks where nucleotide compositional deviations occur. In the bottom part of the figure the nucleotide sequence of predicted oriC region is shown. Open rectangles show the dnaA and dnaN genes flanking the ori site with arrows indicating the direction of transcription. Arrows show positions of the putative DnaA binding sites, predicted consensus sequence of the DnaA box is shown above. (DOC) [file pone.0053047.s001.doc]

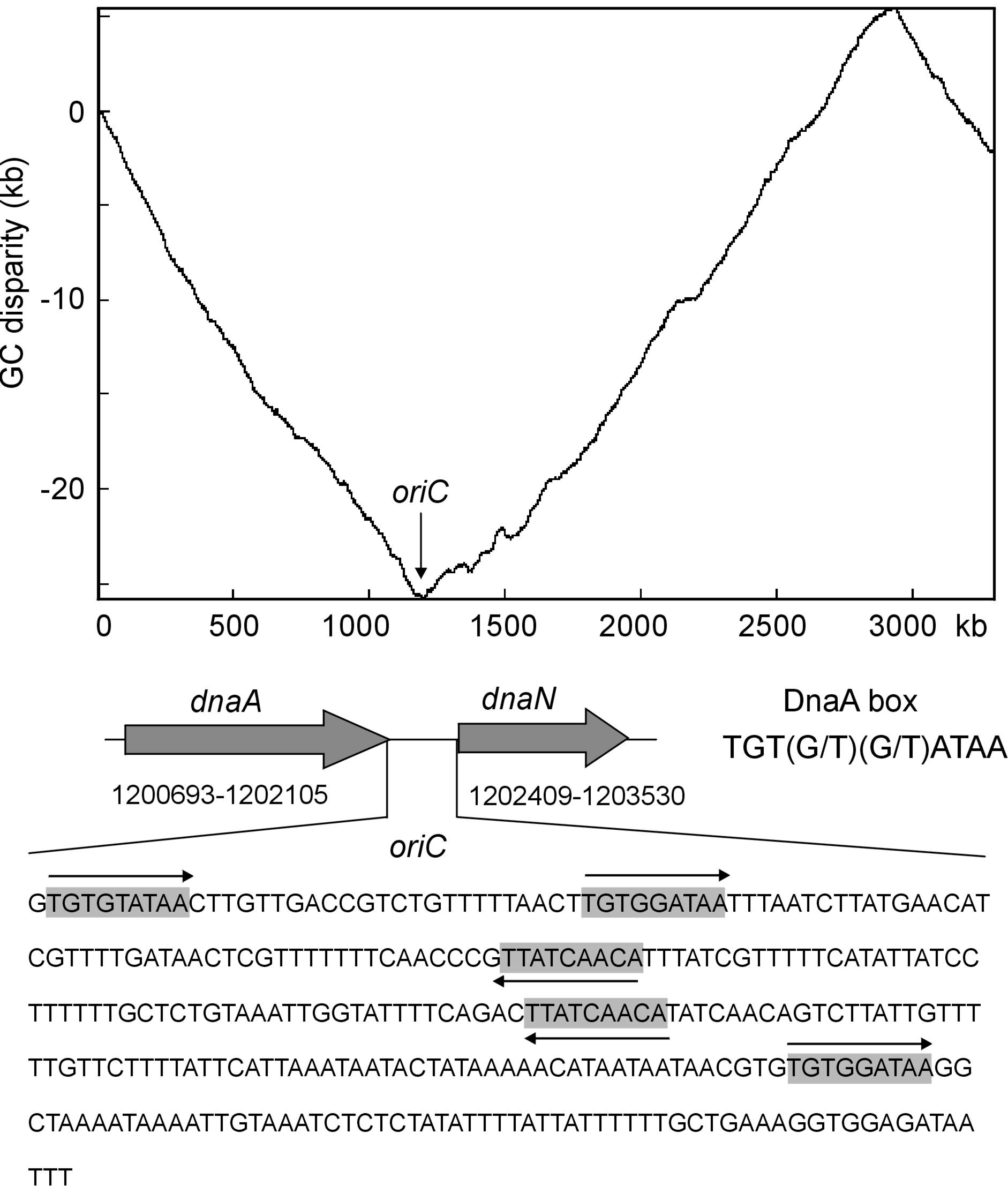


Figure S1. A GC skew analysis of the *M. roseus* genome showing the major peaks where nucleotide compositional deviations occur. In the bottom part of the figure the nucleotide sequence of predicted *oriC* region is shown. Open rectangles show the *dnaA* and *dnaN* genes flanking the *ori* site with arrows indicating the direction of transcription. Arrows show positions of the putative DnaA binding sites, predicted consensus sequence of the DnaA box is shown above.
