# Supplementary material for: Genomic Analysis of Melioribacter roseus, Facultatively Anaerobic Organotrophic Bacterium Representing a Novel Deep Lineage within Bacteriodetes/Chlorobi Group
Source: PLoS One. 2013 Jan 2;8(1):e53047. doi: 10.1371/journal.pone.0053047 (PMC3534657; doi:10.1371/journal.pone.0053047)
Supplement: Figure S2 — Maximum likelihood tree based on the concatenation of 39 ribosomal proteins. The ribosomal proteins from Aquifex aeolicus VF5 were used as an outgroup. Numbers at nodes represent bootstrap values (100 replications of the original dataset). The scale bar represents the average number of substitutions per site. The following species of Proteobacteria and Firmicutes were used to construct the tree: Alkalilimnicola ehrlichii MLHE-1 (NC_008340), Anaerococcus prevotii DSM 20548 (NC_013171), Anaeromyxobacter dehalogenans 2CP-C (NC_007760), Aromatoleum aromaticum EbN1 (NC_006513), Bacillus weihenstephanensis KBAB4 (NC_010184), Bartonella quintana str. Toulouse (NC_005955), Bordetella pertussis Tohama I (NC_002929), Bradyrhizobium japonicum USDA 110 (NC_004463), Caldicellulosiruptor bescii DSM 6725 (NC_012034), Campylobacter jejuni subsp. jejuni NCTC 11168 (NC_002163), Desulfitobacterium hafniense Y51 (NC_007907), Desulfohalobium retbaense DSM 5692 (NC_013223), Desulfomicrobium baculatum DSM 4028 (NC_013173), Dichelobacter nodosus VCS1703A (NC_009446), Erythrobacter litoralis HTCC2594 (NC_007722), Ferrimonas balearica DSM 9799 (NC_014541), Haliangium ochraceum DSM 14365 (NC_013440), Halothermothrix orenii H 168 (NC_011899), Halothiobacillus neapolitanus c2 (NC_013422), Helicobacter pylori 26695 (NC_000915), Herminiimonas arsenicoxydans (NC_009138), Listeria innocua Clip11262 (NC_003212), Magnetospirillum magneticum AMB-1 (NC_007626), Mahella australiensis 50-1 BON (NC_015520), Nautilia profundicola AmH (NC_012115), Neisseria meningitidis MC58 (NC_003112), Oenococcus oeni PSU-1 (NC_008528), Staphylococcus aureus subsp. aureus N315 (NC_002745), Thermoanaerobacter italicus Ab9 (NC_013921), Veillonella parvula DSM 2008 (NC_013520). (DOC) [file pone.0053047.s002.doc]

**Deltaproteobacteria**

**Epsilonproteobacteria**

**Alphaproteobacteria**

**Betaproteobacteria**

**Gammaproteobacteria**

**Firmicutes**

Pelodictyon phaeoclathratiforme BU-1 (NC011060)

Chlorobium chlorochromatii CaD3 (NC007514)

Chlorobium luteolum DSM 273 (NC007512)

Chlorobium phaeovibrioides DSM 265 (NC009337)

Chlorobium limicola DSM 245 (CP001097)

Chlorobium tepidum TLS (NC002932)

Chlorobaculum parvum NCIB 8327 (NC011027)

Prosthecochloris aestuarii DSM 271 (NC011059)

Chlorobium phaeobacteroides BS1 (NC010831)

Chloroherpeton thalassium ATCC 35110 (NC011026)

**Chlorobi**

Melioribacter roseus P3M-2 (CP003557)

Ignavibacterium album JCM 16511 (CP003418)

**Ignavibacteriae**

Rhodothermus marinus DSM 4252 (CP001807)

Salinibacter ruber DSM 13855 (NC007677)

Chitinophaga pinensis DSM 2588 (NC013132)

Haliscomenobacter hydrossis DSM 1100 (NC015510)

Flavobacterium psychrophilum JIP02/86 (NC009613)

Gramella forsetii KT0803 (NC008571)

Cellulophaga lytica DSM 7489 (NC015167)

Fluviicola taffensis DSM 16823 (NC015321)

Porphyromonas gingivalis W83 (NC002950)

Bacteroides thetaiotaomicron VPI-5482 (NC004663)

Prevotella ruminicola 23 (NC014033)

Pedobacter heparinus DSM 2366 (NC013061)

Sphingobacterium spiritivorum ATCC 33861 (ACHA00000000)

Cyclobacterium marinum DSM 745 (NC015914)

Leadbetterella byssophila DSM 17132 (NC014655)

Cytophaga hutchinsonii ATCC 33406 (NC008255)

**Bacteroidetes**

Caldithrix abyssi DSM 13497 (NZCM001402)

Fibrobacter succinogenes subsp succinogenes S85 (NC013410)

Aquifex aeolicus VF5 (NC00918)

100

98

100

100

100

98

100

90

100

100

86

100

65

100

99

100

100

100

100

100

100

100

91

100

100

100

100

89

96

100

90

100

74

100

54

100

100

100

100

0.1

**Figure S2. Maximum likelihood tree based on the concatenation of 39 ribosomal proteins**.  The ribosomal proteins from *Aquifex aeolicus* VF5 were used as an outgroup. Numbers at nodes represent bootstrap values (100 replications of the original dataset). The scale bar represents the average number of substitutions per site. The following species of Proteobacteria and Firmicutes were used to construct the tree: *Alkalilimnicola ehrlichii* MLHE-1 (NC_008340), *Anaerococcus prevotii* DSM 20548 (NC_013171), *Anaeromyxobacter dehalogenans* 2CP-C (NC_007760), *Aromatoleum aromaticum* EbN1 (NC_006513), *Bacillus weihenstephanensis* KBAB4 (NC_010184), *Bartonella quintana* str. Toulouse (NC_005955), *Bordetella pertussis* Tohama I (NC_002929), *Bradyrhizobium japonicum* USDA 110 (NC_004463), *Caldicellulosiruptor bescii* DSM 6725 (NC_012034), *Campylobacter jejuni* subsp. jejuni NCTC 11168 (NC_002163), *Desulfitobacterium hafniense* Y51 (NC_007907), *Desulfohalobium retbaense* DSM 5692 (NC_013223), *Desulfomicrobium baculatum* DSM 4028 (NC_013173), *Dichelobacter nodosus* VCS1703A (NC_009446), *Erythrobacter litoralis* HTCC2594 (NC_007722), *Ferrimonas balearica* DSM 9799 (NC_014541), *Haliangium ochraceum* DSM 14365 (NC_013440), *Halothermothrix orenii* H 168 (NC_011899), *Halothiobacillus neapolitanus* c2 (NC_013422), *Helicobacter pylori* 26695 (NC_000915), *Herminiimonas arsenicoxydans* (NC_009138), *Listeria innocua* Clip11262 (NC_003212), *Magnetospirillum magneticum* AMB-1 (NC_007626), *Mahella australiensis* 50-1 BON (NC_015520), *Nautilia profundicola* AmH (NC_012115), *Neisseria meningitidis* MC58 (NC_003112), *Oenococcus oeni* PSU-1 (NC_008528), *Staphylococcus aureus* subsp. aureus N315 (NC_002745), *Thermoanaerobacter italicus* Ab9 (NC_013921), *Veillonella parvula* DSM 2008 (NC_013520).
