# Supplementary material for: Genomic Analysis of Melioribacter roseus, Facultatively Anaerobic Organotrophic Bacterium Representing a Novel Deep Lineage within Bacteriodetes/Chlorobi Group
Source: PLoS One. 2013 Jan 2;8(1):e53047. doi: 10.1371/journal.pone.0053047 (PMC3534657; doi:10.1371/journal.pone.0053047)
Supplement: Table S1 — Carbohydrate-active enzymes encoded by the M. roseus genome. (DOC) [file pone.0053047.s003.doc]

Table S1. Carbohydrate-active enzymes encoded by the *M. roseus* genome.

| CAZY Family | Gene | CBM Family | Signal peptide | In silico prediction |
| --- | --- | --- | --- | --- |
| Glycoside Hydrolases | | | | |
| GH1 | Mros0638 |  | no | β-glucosidase |
| GH2 | Mros0082 |  | no | β-galactosidase |
| GH2 | Mros0084 |  | no | β-galactosidase |
| GH2 | Mros0505 |  | yes | β-galactosidase |
| GH2 | Mros0564 |  | yes | β-glucuronidase |
| GH2 | Mros0565 |  | yes | β-glucuronidase |
| GH2 | Mros0969 |  | yes | β-galactosidase |
| GH2 | Mros0971 |  | yes | β-galactosidase |
| GH2 | Mros0978 |  | yes | β-galactosidase |
| GH2 | Mros0984 |  | yes | β-galactosidase |
| GH2 | Mros1294 |  | no | β-mannosidase |
| GH2 | Mros1645 |  | yes | β-galactosidase |
| GH2 | Mros1842 |  | yes | β-galactosidase |
| GH2 | Mros2081 |  | yes | β-galactosidase |
| GH2 | Mros2320 |  | no | β-galactosidase |
| GH2 | Mros2388 |  | yes | β-galactosidase |
| GH3 | Mros0754 |  | yes | β-glucosidase |
| GH3 | Mros1580 |  | yes | β-glucosidase |
| GH3 | Mros2076 |  | no | β-glucosidase |
| GH3 | Mros2084 |  | yes | β-glucosidase |
| GH3 | Mros2204 |  | yes | β-glucosidase |
| GH5 | Mros0504 |  | yes | endoglucanase |
| GH5 | Mros0511 |  | yes | mannanase |
| GH5 | Mros0753 |  | yes | endoglucanase |
| GH5 | Mros0960 |  | yes | endoglucanase |
| GH5 | Mros1693 | CBM6 | yes | endo-β-1,4-mannosidase |
| GH5 | Mros2625 |  | no | endoglucanase |
| GH9 | Mros0757 |  | yes | endoglucanase |
| GH9 | Mros2241 |  | yes | endoglucanase |
| GH9 | Mros2626 |  | no | endoglucanase |
| GH9 | Mros2837 |  | yes | endoglucanase |
| GH10 | Mros2091 | CBM4/9 | yes | xylanase |
| GH10 | Mros2495 |  | yes | xylanase |
| GH13 | Mros0648 | CBM48 | no | pullulanase/alpha amylase |
| GH13 | Mros0758 | CBM48 | yes | α-amylase |
| GH13 | Mros0762 |  | yes | α-amylase |
| GH16 | Mros0114 |  | no | β-glucanase |
| GH16 | Mros0976 |  | yes | β-glucanase |
| GH20 | Mros1591 |  | yes | β-hexosaminidase |
| GH26 | Mros0510 |  | yes | β-mannanase |
| GH26 | Mros2623 |  | yes | β-mannanase |
| GH28 | Mros0073 |  | no | polygalacturonase |
| GH28 | Mros2351 |  | no | exo-polygalacturonosidase |
| GH28 | Mros2607 |  | no | polygalacturonase |
| GH29 | Mros1589 |  | yes | α-L-fucosidase |
| GH29 | Mros1590 |  | no | α-L-fucosidase |
| GH29 | Mros2103 |  | no | α-L-fucosidase |
| GH30 | Mros0117 |  | yes | glucosylceramidase |
| GH30 | Mros0981 |  | no | xylanase |
| GH30 | Mros2090 |  | yes | glucuronoarabinoxylan endo-1,4-beta-xylanase |
| GH31 | Mros0086 |  | yes | α-glucosidase |
| GH31 | Mros0763 |  | yes | α-glucosidase |
| GH31 | Mros1644 |  | yes | α-glucosidase |
| GH31 | Mros2611 |  | no | α-galactosidase |
| GH35 | Mros0967 |  | no | β-galactosidase |
| GH42 | Mros0970 |  | yes | β- agarase |
| GH43 | Mros0968 |  | no | β-glucanase |
| GH43 | Mros2087 |  | no | β-xylosidase |
| GH43 | Mros2088 | CBM6 | yes | β-xylosidase |
| GH43 | Mros2093 |  | yes | α-L-arabinofuranosidase |
| GH43 | Mros2345 |  | yes | β-xylosidase |
| GH47 | Mros1586 |  | yes | α-mannosidase |
| GH51 | Mros0980 |  | yes | α-L-arabinofuranosidase |
| GH51 | Mros1708 |  | yes | α-L-arabinofuranosidase |
| GH53 | Mros0961 |  | yes | arabinogalactan endo-1,4-beta-galactosidase |
| GH67 | Mros2496 |  | yes | α-glucuronidase |
| GH77 | Mros2201 |  | no | 4-α-glucanotransferase |
| GH88 | Mros0077 |  | yes | unsaturated rhamnogalacturonyl hydrolase |
| GH88 | Mros0080 |  | yes | unsaturated β-glucuronyl hydrolase |
| GH88 | Mros0977 |  | yes | unsaturated β-glucuronyl hydrolase |
| GH88 | Mros1056 |  | no | unsaturated β-glucuronyl hydrolase |
| GH88 | Mros2319 |  | no | unsaturated β-glucuronyl hydrolase |
| GH88 | Mros2349 |  | yes | unsaturated β-glucuronyl hydrolase |
| GH88 | Mros2624 |  | no | unsaturated β-glucuronyl hydrolase |
| GH92 | Mros1594 |  | yes | α-1,2-mannosidase |
| GH97 | Mros1490 |  | yes | α-glucosidase |
| Carbohydrate Esterases | | | | |
| CE8 | Mros0879 |  | yes | pectin methylesterase |
| CE8 | Mros2353 |  | yes | pectin methylesterase |
| Polysaccharide Lyases | | | | |
| PL1 | Mros0560 | CBM35 | yes | pectate lyase |
| PL1 | Mros2342 |  | yes | pectate lyase |
| PL9 | Mros0085 |  | yes | pectate lyase |
| PL10 | Mros1505 |  | yes | pectate lyase |
| PL10 | Mros2352 |  | no | pectate lyase |
| PL11 | Mros0087 | CBM35 | no | rhamnogalacturonan lyase |
| PL6 | Mros1361 |  | yes | alginate lyase |
| Carbohydrate Binding Modules * | | | | |
|  | Mros0583 | CBM4/9 | no | carbohydrate binding module |
|  | Mros0752 | CBM4/9 | yes | carbohydrate binding module |
|  | Mros1059 | CBM48 | no | carbohydrate binding module |

* proteins without detectable catalytic domains
